# A multidimensional framework for mapping social need to electronic health records in people with multimorbidity

**DOI:** 10.1038/s41598-025-34881-9

**Published:** 2026-01-27

**Authors:** Tassella Isaac, Glenn Simpson, Lucy Smith, Alim Sabary, Nazrul Islam, Miriam Santer, Andrew Farmer, Hajira Dambha-Miller

**Affiliations:** 1 Primary Care Research Centre, University of Southampton, Southampton, SO16 5ST UK; 2 Nuffield Department of Primary Care Health Sciences, University of Oxford, Oxford, UK

**Keywords:** Multimorbidity, Social needs, Integrated care, Electronic health records, Multidimensional framework, Diseases, Health care, Medical research, Risk factors

## Abstract

Social needs are sociocultural and economic factors influencing health and quality of life, including, for example, mobility limitations or financial difficulties. Multimorbidity - the presence of two or more long-term conditions (LTCs) - is an increasing public health challenge, with social needs often compounding the negative health outcomes associated with multimorbidity. In this study, we present a novel multidimensional framework for identifying and characterising social needs within a population-based cohort of adults with multimorbidity in England, utilising data from the Clinical Practice Research Datalink. The framework identifies eight critical domains of social needs: activities of daily living, mobility, financial constraints, disability, community care, housing status, social support, and bereavement. More than 100 related variables were captured in the dataset. Among 7,290,716 individuals with multimorbidity, 36.96% reported at least one social need, with the majority of affected individuals being older, female, and experiencing a higher burden of LTCs. The most prevalent social needs were related to community and social care services. Our findings underscore the significant association between social needs and multimorbidity, revealing a disproportionate burden of social needs in this population. This framework offers a systematic approach to quantifying and measuring social needs, providing a foundation for incorporating these factors into clinical care and interventions.

**Supplementary Information:**

The online version contains supplementary material available at 10.1038/s41598-025-34881-9.

## Introduction

Social needs are crucial determinants of health and quality of life, encompassing a range of sociocultural and economic factors that significantly influence individuals’ well-being^1,2^. For example, these include financial difficulties, housing challenges or problems related to socially connecting with others, which can all hinder participation in daily activities and diminish social and economic engagement. Such social needs exacerbate the risk of frailty, disability, and social isolation, which in turn contribute to poorer health outcomes and elevated mortality risks^2^. Furthermore, these needs often compound the clinical complexities faced by individuals living with long-term conditions (LTCs), creating a multidimensional challenge for healthcare systems^1^.

Multimorbidity, defined as the co-existence of two or more long-term conditions, is becoming an increasingly pressing issue for healthcare systems. In England, the prevalence of multimorbidity among individuals aged over 65 was 54% in 2015, with projections indicating this figure will rise to 67.8% by 2035^3^. Globally, multimorbidity represents a substantial proportion of healthcare demand. It has been shown that multimorbidity accounts for 78% of primary care consultations in high-income countries and is associated with higher hospital admission rates and extended hospital stays^4^. While the clinical management of multimorbidity is well-documented, the social needs of individuals with multimorbidity remain underexplored^5^. Despite growing recognition of the significant impact of these social needs on health, challenges such as inconsistent data coding, unreliable proxy measures, and the narrow lens through which formal social services assess needs have hindered comprehensive research^5–9^. The increasing availability of large-scale electronic health records (EHR), which systematically capture a wide range of social need variables across the life course, presents a valuable opportunity to address these gaps and offer a more nuanced understanding of social needs in the context of multimorbidity. There is currently an absence of standardised guidance or established coding frameworks for the systematic mapping and categorisation of social needs in large-scale electronic health record datasets, particularly in relation to multimorbidity populations both in the UK and globally^10–12^. Most existing work in this field has primarily focused on qualitative checklists or structured reporting frameworks^13^. These provide limited guidance or insight into the practical methodologies for identifying, extracting and curating relevant and comprehensive data, which can be used to analyse and understand social needs in multimorbidity populations. Building on earlier work, this study contributed to finalising the development of a comprehensive, multidimensional framework designed to systematically identify and characterise social needs within EHR. Using this framework, we explore the prevalence and distribution of social needs among 7,290,716 adults with multimorbidity in England. We quantify the association between social needs and long-term conditions across demographic groups, providing critical insights into the complex interplay between social and clinical factors in multimorbidity.

## Methods

### Design of a multidimensional framework to map social needs in electronic health records

**Fig. 1:**
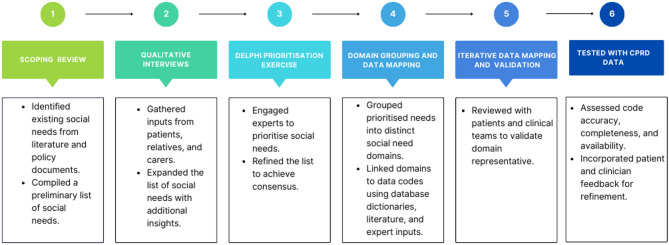
Workflow for developing and grouping social needs by domain using electronic health records.

The framework was developed using a structured, multidimensional ontological process incorporating mixed methods. The development of the multidimensional framework to map social needs in EHR followed a structured iterative six-stage process (Fig. 1), combining both previously published and novel methodological components. While stages 1–3 have been reported in separate publications^7,9,14^, this earlier work was conducted to generate and refine the list of relevant social needs, grounding the entire multidimensional framework in both empirical evidence and lived experience. These stages provided the foundational content, essential to the current study. Stages 4–6 represent the novel methodological contributions of this study, in which the framework was structured, mapped to EHR codes, and tested in large-scale datasets.

### Stage 1: scoping review

The first stage involved a review to identify both published and grey literature on social needs in the context of EHR and multimorbidity^9^. The primary goal of this phase was to gather evidence on how social needs are captured and coded in large primary care datasets. In the UK and elsewhere in the world, multimorbidity is primarily managed within the primary care sector and as a result, it is likely that a substantial amount of social need in the multimorbidity population will be recorded in primary care health records^9^. The results of the review produced an initial list of clinically relevant social needs for populations with multimorbidity and provided valuable insights that guided the subsequent conceptualisation of social needs within the EHR framework development.

### Stage 2: qualitative interviews

Building on the findings from the scoping review, the second stage involved 29 semi-structured interviews with people who have multimorbidity, their carers, and healthcare professionals. These interviews offered in-depth perspectives on social needs from those directly impacted by multimorbidity, ensuring that the framework was grounded in lived experiences and real-world care contexts. The qualitative insights helped to identify additional social needs, which were integrated with the initial list, further refining and advancing the framework’s conceptual development^14^.

### Stage 3: delphi prioritisation exercise

To refine the framework further, a Delphi prioritisation exercise was conducted with 20 key stakeholders, including clinicians, social care professionals, data scientists, members of the public, people living with multimorbidity and their carers. The purpose of this exercise was to prioritise the social needs identified in the previous stages. Through three iterative rounds, consensus was reached on 12 priority social needs that would be mapped into electronic health records, which became the conceptual basis for the framework. This process ensured that the framework was not only evidence-based but also aligned with the perspectives of those with expertise across clinical, social care, and patient contexts, thereby strengthening its practical relevance for multimorbidity populations^7^.

### Novel contributions of this work (stages 4–6)

The specific methods developed and refined in this study (stages 4–6) are underpinned by the findings from the mixed-methods empirical work in stages 1–3. We describe this methodological development below.

### Stage 4: domain grouping and data mapping

In the current study, the list of priority social needs was categorised into eight ‘domains’ after a number of iterations by a multidisciplinary research team, consisting of qualitative and quantitative researchers, clinicians and social care professionals, and public and patient representatives. This work focused on achieving alignment with clinical and social care practice and research priorities. Each domain was carefully structured and named to encapsulate the identified social needs comprehensively. This was an essential conceptual step in transforming a broad list of social needs into an operational framework to enable structured data mapping in the EHR. Table 1 provides detailed descriptions of each domain.

### Stage 5: iterative data mapping and validation

Following the methodological work conducted in stage 4, the established framework was systematically applied to two large EHR datasets, specifically the Clinical Practice Research Datalink (CPRD) GOLD and Aurum databases^15^. Each social need domain was carefully mapped to corresponding SNOMED and READ codes, ensuring mutual exclusivity between the domains. This stage was important as it provided validation that the proposed social need domains could be reliably identified within existing EHR datasets. Feedback was gathered from people living with multimorbidity, carers and care professionals through multiple validation cycles, refining the framework to ensure its relevance and applicability to real-world care practices.

### Stage 6: testing with CPRD data

In the final phase, the framework was tested using data from the large CPRD datasets. This testing phase aimed to confirm the practical utility of the framework at scale when applied to extensive electronic health records. The results demonstrated the framework’s effectiveness in identifying, categorising, and mapping a wide range of social needs, proving its potential for enhancing both research and care management for populations with multimorbidity.

Taken together, these stages were methodologically aligned with the study’s goal of creating a framework that is not only conceptually sound but also can be operationalised in primary care EHR datasets.

Using the derived multidimensional framework to describe the distribution of social need and quantify the association between social need and long-term conditions.

### Study population and data source

Data from the Clinical Practice Research Datalink (CPRD) GOLD and AURUM databases were used, which include anonymised primary care records linked to hospital and socioeconomic data. The dataset covers over 16 million registered patients, representative of England’s population by age, sex, ethnicity, and Index of Multiple Deprivation (IMD)^16^. Adults aged ≥ 18 years with multimorbidity (defined as two or more chronic conditions) were included, covering data from January 1, 1987, to December 31, 2020^17^. The index date was defined as the earliest date on which an individual’s second qualifying LTC was recorded; age was calculated at the index date. Each individual contributed one observation only, identified by a unique patient identifier. Geographic region was defined based on the postcode of the patient’s registered general practice and mapped to one of the standard NHS administrative regions in England. Nine out of the 13 standard regions were included, selected based on the study’s focus and population coverage.

### Defining multimorbidity

Multimorbidity was defined as the presence of two or more long-term conditions (LTCs). LTCs were conceptualised as ‘health problems that require ongoing management over a period of years or decades and cannot currently be cured but can be controlled with the use of medication and/or other therapies’, as defined by the NHS Data Model and Dictionary (2024)^18^.

A list of 59 long-term conditions (LTCs) was developed through a national consensus process involving multimorbidity stakeholders and researchers from the United Kingdom^19^. Of these, 54 conditions were included in this analysis, grouped based on data availability within CPRD. The full list of included conditions, along with their corresponding codes, is publicly available on Git-Hub. Additional information on the conditions included can be found in Supplementary Tables 1, and a full list of diagnostic READ and SNOMED codes used to define each LTC is available in Supplementary Table 2.

### Statistical analysis

Variables related to social needs within CPRD were classified into the eight derived domains (Table 1) and analysed to evaluate their prevalence and associations with multimorbidity. This structure enabled the assessment of the following associations: (i) the relationship between the presence of multimorbidity and social needs, and (ii) the correlation between the number of social needs and the count of long-term conditions (LTCs). Demographic characteristics, including age, sex, ethnicity, IMD, and region, were summarised for the overall multimorbidity cohort and for those with at least one reported social need. A full list of the specific codes used to define each domain is provided in Supplementary Table 3. Social need codes were considered regardless of when they were recorded; accordingly, the domain indicators and summary measures reflect cumulative recorded social need history.

Logistic regression models were used to assess the associations between the number of LTC and the presence of any social need, as well as the eight specific social need domains, with social need modelled as a binary outcome. Linear regression models were used to evaluate the number of LTCs as a predictor of the total number of social needs. We adjusted for sociodemographic variables in all the regression models. Missing data were handled using prespecified rules. Social need variables were coded as binary (1 = present, 0 = not recorded), and unrecorded fields were treated as 0 when deriving the “any social need” indicator and the total social need count. For all regression analyses, we used complete-case evaluation for the model covariates (age, LTC count, sex, ethnicity, IMD, and region). A two-sided p-value < 0.05 was considered statistically significant for all analyses.

**Table 1:** Domains and descriptions of social needs as defined in the framework. (CPRD GOLD and Aurum, England, 1987–2020).

Domain	Description
Activities of daily living needs	Social needs associated with carrying out activities of daily living (ADLs) including: • Ambulating - the extent of an individual’s capability to move from one position to another, perform basic movements and dexterity. • Feeding - ability to feed oneself without assistance. • Dressing - ability to put on their clothes and select appropriate clothing. • Personal hygiene - ability to undertake basic washing, bathing, and grooming, maintain personal hygiene including dental, nail, and hair care. • Continence – ability to control bladder and/or bowel function. • Toileting - ability to get to and from the toilet, using it appropriately and cleaning oneself.
Mobility needs	Social needs resulting from difficulties with physical mobility, require the provision of mobility aids or equipment, professional input and care and financial support with mobility including: • Wheelchair use. • Non-wheelchair mobility aid use (e.g., walking stick, Zimmer frame, mobility scooter). • Professional care staff provision to assist with mobility. • Receives disability living allowance. • Receives mobility allowance.
Financial needs	Social needs relating to challenges an individual may have managing their personal finances employment-related difficulties and receiving assistance from a range of financial support services, including: • Difficulty budgeting and handling money. • Employment status (e.g., unemployed, or long-term sick). • Referral and/or input from financial adviser or financial services. • Receipt of assistance from support services such as food banks, accessing the affordable warm programme. • Difficulty writing cheques, using a credit cards, carrying out arithmetic reasoning related to money and difficulty managing bank accounts.
Disability needs	Social needs resulting from specific disabilities or impairments including: • Mental health disability. • Intellectual disability. • Learning disability. • Speech, hearing, and sensory disabilities. • Any state assessment of disability.
Community care needs	Social needs requiring support from a range of community health and social care services and practitioners, including: • Community physiotherapist or occupational therapy. • Drug and alcohol team. • Mental health team. • Social care services. • Outreach or voluntary services. • Community nurses, specialist nurses or matrons. • Community secondary care professionals. • Audiology. • Palliative care team. • Dietician. • Community care navigator or social care prescribers. • Pharmacy input.
Residency status needs	Social needs relating to residential status including: • Hospice care. • Nursing home care. • Care home. • Own home with adaptations.
Social care networking needs	Social care needs associated with an individual’s ability to socially connect, network, and participate in social care activities including: • Ability to maintain meaningful relationships with family, friends, and others. • Ability to socialise and mix with others in social care contexts. • Ability to effectively communicate verbally and non-verbally. • Ability to participate in hobbies, leisure, and community activities. • Access to and ability to organise transportation. • Input from professional services to socially connect and participate such as day units or visits from charities for loneliness.
Bereavement needs	Social care needs arising from family bereavement including: • Death of a partner, husband, wife, sibling, child including neonatal and postnatal death, sudden infant death, maternal death. • Input from professional services including referral to or use of bereavement counselling or therapies.

All analyses were conducted in R (version 4.2.1).

### Ethics approval and consent to participate

This study used anonymised routinely collected primary care electronic health record data from the Clinical Practice Research Datalink (CPRD) Gold and Aurum databases. Ethical approval was granted by the University of Southampton Faculty of Medicine Research Ethics Committee (reference 67953). The study was also approved by the Independent Scientific Advisory Committee for CPRD (ISAC protocol 21_001667). Primary care practices provide consent for CPRD to collect de-identified data from their practice. Individual patients can opt out of sharing their data for research and the data providers do not collect data for those patients. All methods were performed in accordance with relevant guidelines and regulations, including the Declaration of Helsinki. As this study used de-identified routinely collected data, the requirement for written informed consent from individual patients was waived by the University of Southampton Faculty of Medicine Research Ethics Committee (reference 67953) and the Independent Scientific Advisory Committee (ISAC protocol 21_001667), in line with CPRD governance procedures.

## Results

### Mapping the framework to CPRD data

**Fig. 2:**
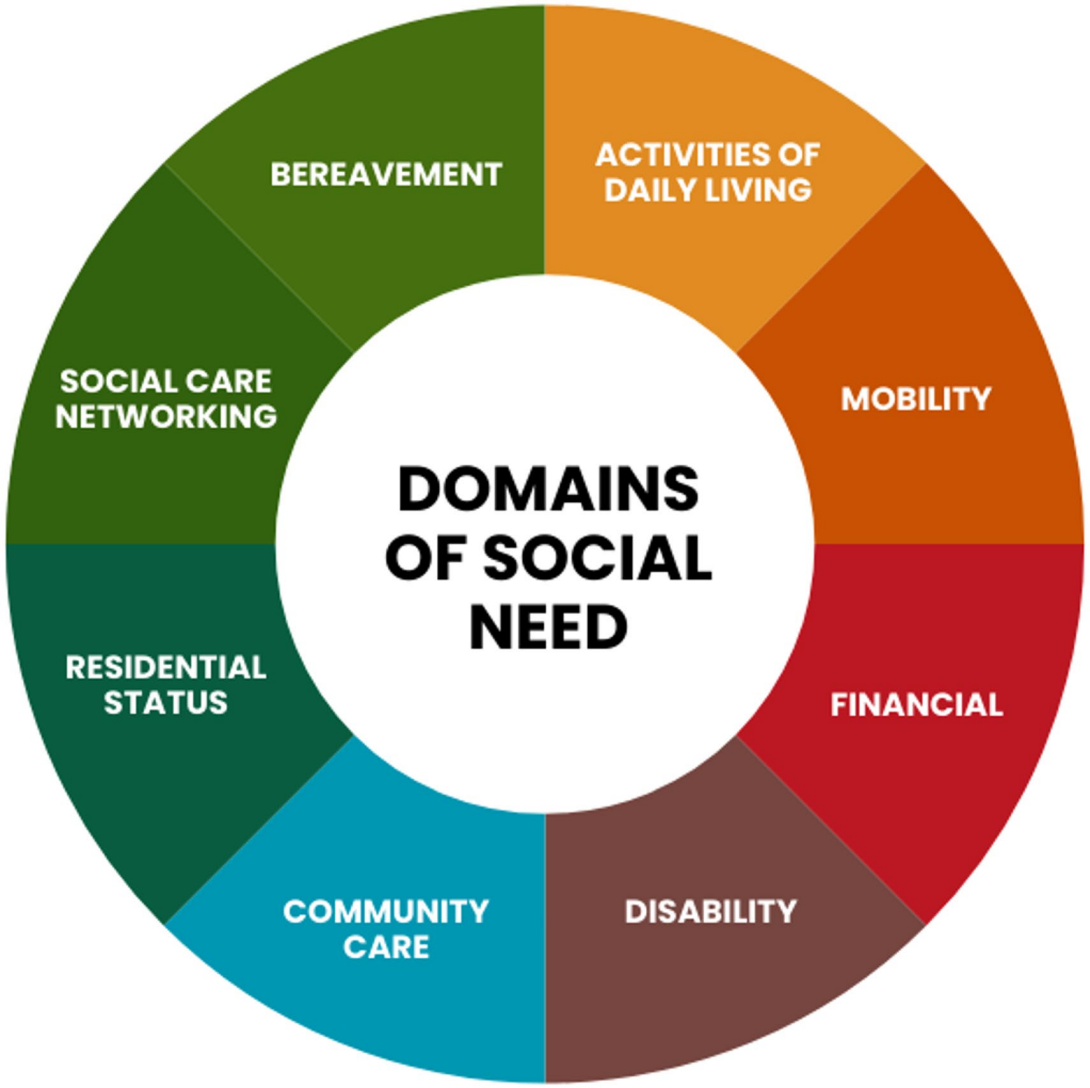
The eight social need domains derived from the framework.

In this study eight distinct social need domains were identified and defined as: (1) Activities of Daily Living (ADL), (2) Mobility Needs, (3) Financial Needs, (4) Disability Needs, (5) Community Care Needs, (6) Residential Status Needs, (7) Social Care Networking Needs, and (8) Bereavement Needs. These domains are schematically represented in Fig. 2. Detailed descriptions of each domain and the associated indicators used in the data mapping are provided in Table 1. The framework methodology was successfully applied to primary care EHR data from the CPRD GOLD and Aurum datasets, and the results are presented below.

Framework-derived definitions of the eight social-need domains used in this study: Activities of daily living, Disability, Community care, Mobility, Residential status, Social care networking, Bereavement, and Financial—operationalised from routinely collected primary-care records in CPRD GOLD and Aurum (1987–2020). For each domain, the table lists a brief description and the electronic health record constructs used.

### Demographic characteristics

The cohort comprised 7,290,716 individuals with multimorbidity, of whom 37% (2,694,488 individuals) reported at least one social need, and 15.8% (1,148,247 individuals) reported two or more social needs. Individuals with social needs tended to be older, with an average age of 77 years, compared to an average of 70 years for those without social needs. Additionally, a higher proportion of individuals with social needs were female, accounting for 57.2% of this group, compared to 55.3% among those without social needs.

**Fig. 3:**
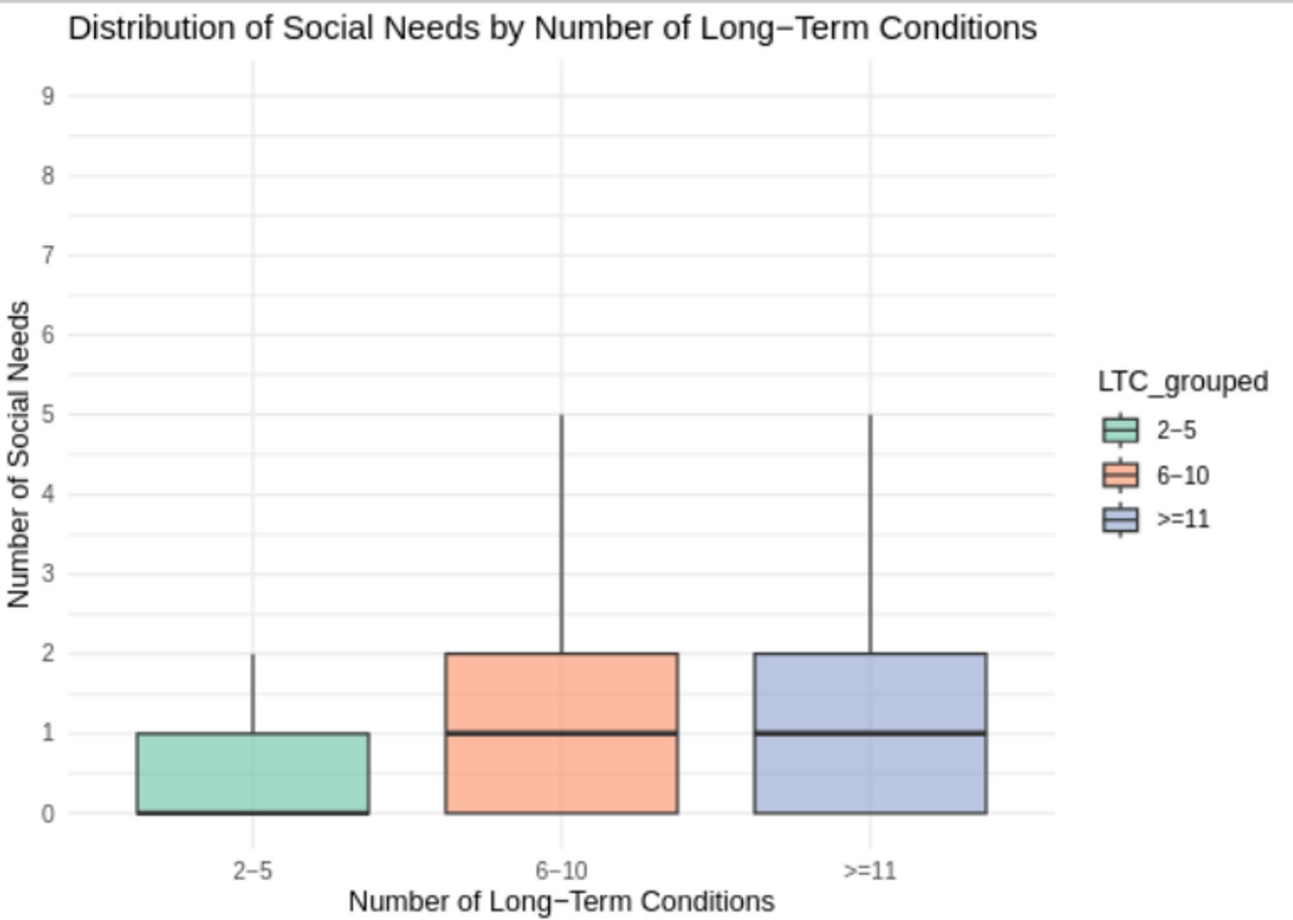
Distribution of social-need counts by number of long-term conditions in CPRD GOLD and Aurum (England, 1987–2020).

Regional variation was also evident in the frequency of social needs, with the highest proportions reported in the Northwest (20.4%), South Central (19.9%) and West Midlands (16%). Additionally, the mean number of long-term conditions (LTCs) was higher among those with social needs (6.35 vs. 4.53), and they were more likely to report 10 or more LTCs (11.6% vs. 3.2%), comparing individuals with and without social needs. These demographic characteristics are summarised in Table 2. The distribution of social needs across different levels of long-term conditions is shown in Fig. 3, with the median value represented for each group (1–5, 6–10, and 11–15).

**Table 2:** Sociodemographic characteristics of adults with multimorbidity in CPRD GOLD and aurum (England, 1987–2020), stratified by ≥ 1 recorded social need.

Characteristic	Total cohort (*n* = 7,290,716)	Cohort with social needs (*n* = 2,694,488)	Cohort without social needs (*n* = 4,596,228)
Demographics			
Age statistics			
Mean age, years (SD)	73 (21.57)	77 (20.29)	70 (21.87)
Median age, years	74	80	70
Age range, n (%)			
18–44	2, 195, 538 (30.1)	622, 575 (23.1)	1, 572, 963 (34. 2)
45–64	2, 690, 702 (36.9)	1, 003, 629 (37.2)	1, 687, 073 (36.7)
65–74	1, 257, 871 (17.3)	552, 506 (20.5)	705, 365 (15.3)
75–84	785, 914 (10.8)	365, 759 (13.6)	420, 155 (9.1)
85+	271, 270 (3.7)	119, 371 (4.4)	151, 899 (3.3)
Unknown	89, 421 (1.2)	30, 648 (1.1)	58, 773 (1.3)
Sex			
Male, n (%)	3, 257, 846 (44.7)	1, 154, 279 (42.8)	2, 103, 567 (45.8)
Female, n (%)	4, 032, 764 (55.3)	1, 540, 159 (57.2)	2, 492, 605 (54.2)
Unknown	106	50	56
Index of multiple deprivation (IMD)			
IMD Quintile 1 (Least Deprived), n (%)	1, 397, 456 (19.2)	501, 095 (18.6)	896, 361 (19.5)
IMD Quintile 2, n (%)	1, 454, 975 (20)	526, 396 (19.5)	928, 579 (20.2)
IMD Quintile 3, n (%)	1, 425, 668 (19.6)	525, 734 (19.5)	899, 934 (19.6)
IMD Quintile 4, n (%)	1, 486, 908 (20.4)	548, 168 (20.3)	938, 740 (20.4)
IMD quintile 5 (most deprived), n (%)	1, 511, 105 (20.7)	589, 255 (21.9)	921, 850 (20.1)
Unknown	14, 604 (0.2)	3, 840 (0.1)	10, 764 (0.2)
Ethnicity			
Asian, n (%)	252, 292 (3.5)	102, 883 (3.8)	149, 409 (3.3)
Black, n (%)	158, 483 (2.2)	65, 563 (2.4)	92, 920 (2)
Mixed, n (%)	40, 272 (0.6)	14, 063 (0.5)	26, 209 (0.6)
Other ethnic groups, n (%)	90, 554 (1.2)	31, 710 (1.2)	58, 844 (1.3)
White, n (%)	5, 858, 566 (80.4)	2, 277, 075 (84.5)	3, 581, 491 (77.9)
Unknown, n (%)	890, 549 (12.2)	203. 194 (7.5)	687, 355 (15)
Region			
East midlands, n (%)	199, 878 (2.7)	55, 025 (2)	144, 853 (3.2)
East of England, n (%)	387, 348 (5.3)	157, 668 (5.9)	229, 680 (5)
London, n (%)	964, 715 (13.2)	333, 995 (12.4)	630, 720 (13.7)
Northeast, n (%)	263, 546 (3.6)	103, 398 (3.8)	160, 148 (3.5)
Northwest, n (%)	1, 459, 326 (20)	548, 598 (20.4)	910, 728 (19.8)
South central, n (%)	1, 449, 978 (19.9))	537, 108 (19.9)	912, 870 (19.9)
Southwest, n (%)	1, 129, 947 (15.5)	420, 972 (15.6)	708, 975 (15.4)
West midlands, n (%)	1, 135, 681 (15.6)	430, 714 (16)	704, 967 (15.3)
Yorkshire & the humber, n (%)	296, 645 (4.1)	105, 882 (3.9)	190, 763 (4.2)
Unknown	3, 652 (0.1)	1, 128	2, 524 (0.1)
Number of long-term conditions			
Mean	5.21	6.35	4.53
2–5	4, 603, 975 (63.1)	1, 269, 229 (47.1)	3, 334, 746 (72.6)
6–10	2, 225, 358 (30.5)	1, 112, 269 (41.3)	1, 113, 089 (24.2)
> 10	461, 383 (6.3)	312, 990 (11.6)	148, 393 (3.2)

Distributions of age, sex, ethnicity, socioeconomic deprivation (IMD quintiles), region, and number of long-term conditions (LTCs) are presented for the total cohort (*n* = 7,290,716), with social needs (*n* = 2,694,488), and without social needs (*n* = 4,596,228). Column percentages are shown, and ‘Unknown/Missing’ categories are included.

Social-need counts are summarised within LTC groups 2–5, 6–10, and ≥ 11 in the multimorbidity cohort (*n* = 7,290,716). Medians are labelled within each group to illustrate increasing social-need burden with higher multimorbidity.

### Association between LTC and social needs

**Fig. 4:**
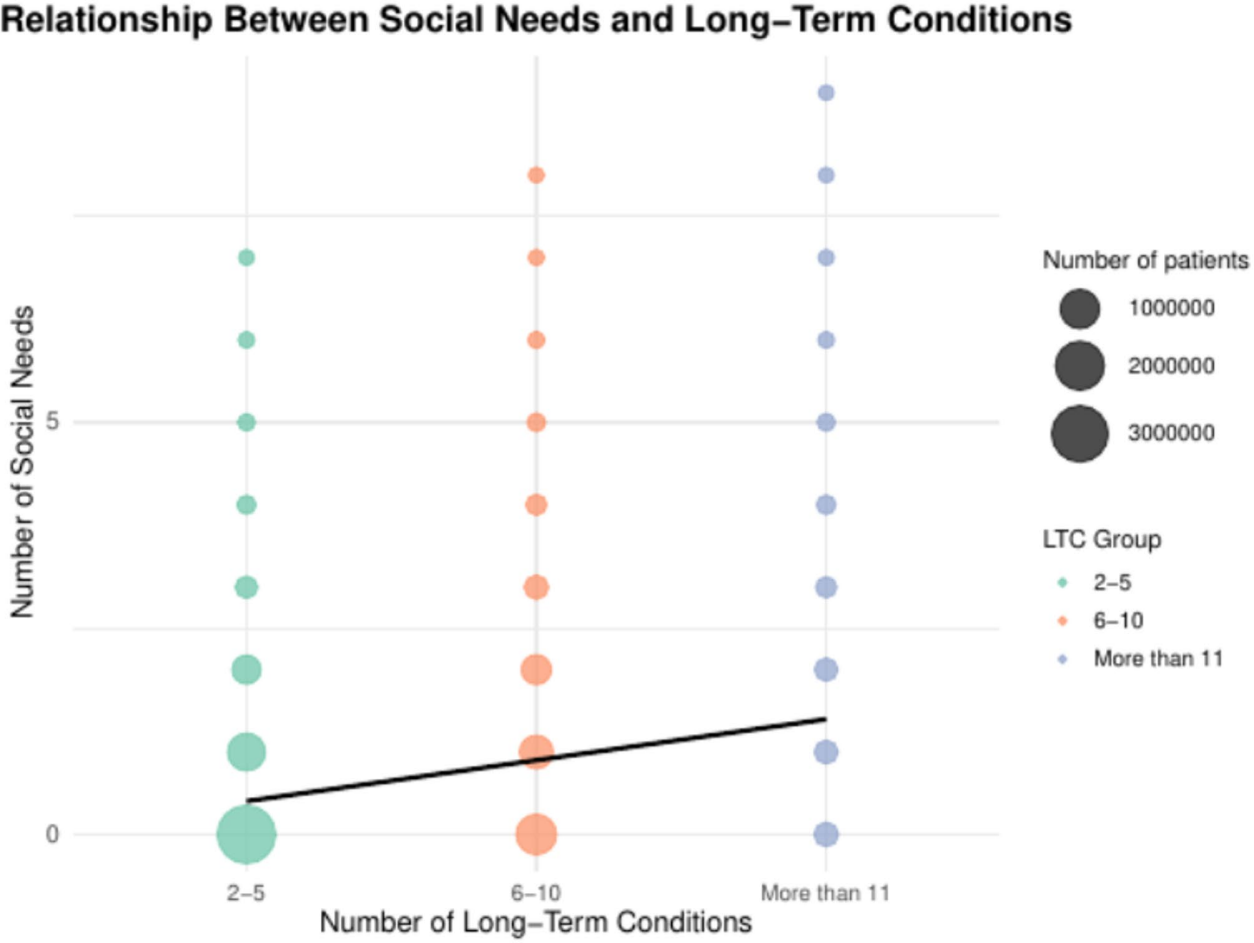
Association between social-need count and number of long-term conditions in CPRD GOLD and Aurum (England, 1987–2020).

There was a statistically significant linear association between the number of LTC and the number of social needs after adjusting for sociodemographic variables, with each additional LTC corresponding to an increase of 0.060 units (95% CI: 0.060–0.061; *p* < 0.001). These findings are visually supported in Fig. 4, which illustrates the positive trend between the number of LTC and the number of social needs.

This figure demonstrates the relationship between social needs (y-axis) and long-term conditions (x-axis), grouped into three categories: 2–5, 6–10, and more than 11 conditions. The size of each data point corresponds to the number of patients within each group, with points color-coded by the number of long-term conditions. A regression line is shown to indicate the overall trend. The analysis reveals a positive correlation between the number of long-term conditions and the number of social needs.

The logistic regression analysis showed a significant association between the number of LTC and the presence of social needs. Each additional LTC was associated with a 24% increase in the odds of having at least one social need in the unadjusted model (OR: 1.24; 95% CI: 1.240–1.241; *p* < 0.001), and 21% in the model adjusted for sociodemographic characteristics (adjusted OR: 1.21; 95% CI: 1.210–1.211 *p* < 0.001).

Domain-specific models revealed that the strongest associations between the number of LTC and individual social need domains were observed for Community care (aOR 1.2013; 95% CI 1.2006–1.2021), Mobility (aOR 1.2005; 95% CI 1.1991–1.2019), and Residential status (aOR 1.1931; 95% CI 1.1920–1.1942). Estimates for other domains were: Disability 1.1780 (95% CI 1.1765–1.1794), Financial 1.1690 (95% CI 1.1669–1.1712), Social care networking 1.1297 (95% CI 1.1287–1.1307), Activities of daily living 1.1078 (95% CI 1.1067–1.1089), and Bereavement 1.0858 (95% CI 1.0841–1.0875) (all *p* < 0.001) (Table 3).

**Table 3:** Association between multimorbidity burden and individual social need domains in CPRD gold and aurum (England, 1987–2020).

Social need domain	Unadjusted OR (95% CI)	Adjusted OR (95% CI)
Activities of daily living	1.1594 (1.1584–1.1605)	1.1078 (1.1067–1.1089)
Disability	1.1608 (1.1605–1.1620)	1.1780 (1.1765–1.1794)
Community care	1.2205 (1.2198–1.2212)	1.2013 (1.2006–1.2021)
Mobility	1.2729 (1.2716–1.2743)	1.2005 (1.1991–1.2019)
Residential status	1.2849 (1.2839–1.2860)	1.1931 (1.1920–1.1942)
Social care networking	1.1563 (1.1554–1.1572)	1.1297 (1.1287–1.1307)
Bereavement	1.1080 (1.1065–1.1095)	1.0858 (1.0841–1.0875)
Financial	1.1538 (1.1519–1.1557)	1.1690 (1.1669–1.1712)

Odds ratios (ORs) per additional LTC are reported for each domain, with unadjusted and adjusted estimates (adjusted for age, sex, ethnicity, IMD, and region).

## Discussion

This study presents a multidimensional framework for identifying social needs within electronic health records and applies it to a cohort of 7.2 million individuals with multimorbidity from the CPRD database. Through this framework, we defined eight domains of social need: activities of daily living, mobility needs, financial needs, disability needs, community care needs, residential status needs, social networking needs, and bereavement support. By applying this framework to a large, nationally representative cohort of individuals with multimorbidity, we successfully identified and mapped a wide range of social need variables, illustrating the strong association between social needs and multimorbidity.

Although conceptually our coding scheme was mutually exclusive, social need domains are likely to overlap in practice and lived experience, in particular, ‘Disability’, ‘Mobility’, ‘Activities of Daily Living’ and ‘Community Care’ and therefore could co-occur and interact to increase or compound need. Therefore, our findings should be interpreted within this context of potentially intersecting domains, and future work is required to refine and strengthen the conceptual clarity of our framework.

Our study shows that 37% of individuals with multimorbidity experience at least one social need, underscoring the significant burden that social needs impose on this population. These findings highlight the complexity of healthcare for individuals with multimorbidity, where clinical management alone is insufficient. Addressing social needs is crucial for improving care outcomes and reducing the overall burden of multimorbidity. The multidimensional nature of these needs means that healthcare systems must integrate both clinical and social care to better support individuals living with multimorbidity. This study validates our framework and demonstrates its feasibility for use in large primary care datasets, providing a valuable foundation for future research on the intersection of social needs and multimorbidity.

Our results are consistent with prior studies, such as Wittchen et al.^20^, which reported that 50% of individuals with multimorbidity experience functional limitations or social care impairments. This study builds upon that evidence by considering a broader spectrum of social needs and exploring their integration into clinical care management. Among individuals with 6–10 long-term conditions, 50% had at least one recorded social need; conversely, 41.3% of those with a social need had 6–10 conditions. In the overall cohort, 30.5% had 6–10 conditions, supporting the interpretation that higher multimorbidity burden is associated with greater social-need prevalence. This finding accords with other studies, which also found a similar association between an individual having a higher number of long-term conditions and more social needs^21–23^. Furthermore, 11.6% of individuals with social needs had more than 10 LTCs, compared to 6.3% in the general cohort, demonstrating the compounded burden of both clinical and social challenges. Similar results have been found in previous research, which have shown this cumulative burden of care needs^24^.

This finding aligns with calls made in earlier research for multifaceted interventions to address these cumulative and interrelated care needs to improve overall care outcomes of patients managing both chronic health conditions and multiple social needs^25^. What is more, failure to address unmet social needs by health and social providers leads to poorer care outcomes for multimorbidity populations. This highlights the need for a care management approach to multimorbidity that encompasses all facets of care needs^26^. The high prevalence of community care needs likely reflects the growing reliance on community-based services, which have been increasingly strained by reductions in home care and domiciliary services due to austerity-related budget constraints and persistent staffing shortages in the sector^27–34^. A recent UK Parliamentary inquiry found that the fragmentation of primary and community care services contributes significantly to the increasing demand for community care^35^. Furthermore, previous research has identified service coordination as a major issue, with both caregivers and healthcare professionals highlighting the need for enhanced integration of clinical and social care services to improve overall care outcomes^36–38^.

Demographically, we observed higher social need prevalence among females and older individuals with multimorbidity, consistent with prior research on the social determinants of health, which show that older women, as well as individuals from ethnic minority and lower socioeconomic backgrounds, experience poorer health outcomes^26,38,39^. These patterns suggest that early targeted interventions addressing both clinical and social needs could help mitigate risks and improve health outcomes for these high-risk groups. By incorporating social needs into healthcare strategies, systems can better manage the complexity of multimorbidity and enhance care outcomes^5,40^. The findings of this study further support the validity and utility of the multidimensional framework for identifying and categorising social needs in multimorbidity populations, highlighting the critical need for a holistic approach to healthcare that integrates both social and clinical factors^41–45^.

Additionally, our logistic regression analysis revealed a significant association between the number of LTCs and the presence of social needs. For each additional LTC, the odds of having at least one social need increased by 24% in the unadjusted model and 21% after adjusting for sociodemographic variables. This further emphasises the compounded burden of social and clinical needs in multimorbidity populations^25^. The linear regression analysis also confirmed a positive correlation between the number of LTCs and the burden of social needs, with each additional LTC contributing to a significant increase in social need burden. These findings highlight the interconnected nature of clinical and social challenges, with individuals experiencing multiple domains of need simultaneously. The strongest associations between multimorbidity burden and social needs were observed for residential, mobility, and community care needs, areas that require significant social support to improve care outcomes. The weaker associations observed in certain social need domains, such as bereavement and social networking, may reflect under-recording in primary care electronic health records (EHRs). These domains are more likely to be recorded in separate third-sector records rather than primary care EHRs, as services for such social needs are often delivered in the community by third-sector organisations. Additionally, bereavement and social networking may not be recognised by care practitioners as formal ‘social needs’, as they may be perceived as representing episodic rather than chronic long-term needs, and therefore these social needs may not be recorded or are under-recorded in primary care EHRs, reflecting the inherent biases in the data captured in primary care EHRs.

While it is not possible to infer direct causality from our analysis, the association between multimorbidity and social needs is likely to be ‘bidirectional’ in nature. First, it is plausible that the accumulation of LTCs increases social needs, particularly in ageing cohorts, as multiple conditions create greater complexity in disease management and interact in ways that intensify patients’ vulnerability and dependency, increasing care demands overall. This has been identified in earlier work, which has highlighted the ‘strong association between multimorbidity and reduced functional capacity’, especially in those individuals with complex multimorbidity (i.e., four or more long-term conditions)^3^. This cohort are also likely to have an increased number of ADL limitations and be at higher risk of falls, etc., requiring more social support.

Second, it is widely recognised that social needs can often be deleterious to health, hasten both disease onset and progression, or exacerbate existing health conditions, especially among individuals living with multiple chronic health conditions. For example, individuals experiencing ‘poor social relationships’ and loneliness are at increased risk of coronary heart disease and stroke, whilst social isolation is associated with ‘early mortality’^46^. Further, poor quality housing has been linked with adverse health outcomes in a number of chronic conditions including respiratory disease, asthma and mental ill-health^47^. Cumulatively, this bidirectional relationship can contribute to poor health outcomes and in doing so, increase health and social needs in multimorbidity populations, especially among the most vulnerable cohorts such as older adults, women, and people in disadvantaged communities.

We recognise that the multidimensional framework presented in this paper is situated within the broader field of social determinants of health models that have been developed over the). last 30 years. While many of these established models provide conceptual frameworks for understanding health inequities more generally, they are often not easily operationalised in practice^48–51^. From a practical standpoint, our work has addressed this issue by demonstrating that our multidimensional framework is effective in identifying, categorising, and mapping a wide range of social needs, including wider social determinants of health, proving its potential for enhancing both research and care management for populations with multimorbidity. Unlike more generic population-scale models, our multidimensional framework is specifically designed and tailored to the complex social needs of multimorbidity populations.

### Strengths and limitations

The primary strength of this study lies in the large, nationally representative sample derived from CPRD, which encompasses a diverse population of individuals with multimorbidity across England. This extensive dataset enabled the inclusion of various variables within each social need domain, providing a robust framework to explore the complex relationship between social needs and multimorbidity. Additionally, the application of a novel, multidimensional approach to capture social needs through electronic health records distinguishes this study, allowing for a more nuanced analysis of social needs across multiple domains. Another strength of our study is that we grouped social need domains using over 100 different variables, which informed the construction of each domain.

Our study should be considered in light of the following limitations. First, the reliance on primary care clinical records may introduce diagnostic misclassification and coding errors, potentially leading to underreporting or misreporting of long-term conditions and social needs. Furthermore, there is evidence that primary care practitioners often experience coding challenges due to the inherent ‘complexity of clinical coding in primary care’, including problems with the operability of systems, time pressures due to clinical workload and difficulties identifying the correct codes^52,53^. While this variation in coding quality could impact the accuracy of our findings, the use of a large, nationally representative dataset from CPRD increases the robustness of the study, particularly for common conditions and social needs. Second, our cohort was skewed towards a higher proportion of females and predominantly White individuals, which may limit the generalisability of the findings to more ethnically diverse populations. As more diverse datasets become available, similar analyses could be performed to provide a broader understanding of social needs across different demographic groups. Third, the observational nature of the study restricts the ability to establish causal relationships between multimorbidity and social needs. While we identified significant associations, the complexity of these relationships warrants further research using longitudinal data or intervention studies to explore causal pathways. Fourth, it is important to recognise that not all social care needs are captured in the primary care clinical record and that there will be a bias towards those needs where there is an overlap with health-related needs or where primary care services need to be engaged to mobilise the social care services to meet those needs. Additionally, we acknowledge that there may be conceptual overlap in practice between some of our social need domains, and therefore, our framework should be interpreted with this possible limitation in mind.

Fifth, our social need measures include historical entries, including some recorded before 1987 or outside a patient’s registration. This maximises capture of lifetime recorded need but may include resolved needs; as a result, social need prevalence may be overestimated and attenuate effect sizes. Estimates should therefore be interpreted as cumulative recorded burden, not point prevalence.

Finally, the framework developed here provides a novel approach for quantifying and measuring social needs within electronic health records, but the general applicability of this framework to other healthcare settings or databases internationally should be validated in future studies to ensure its versatility and scalability.

## Conclusion

In conclusion, we demonstrate the significant association between social needs and multimorbidity in a large, nationally representative cohort. By developing a novel multidimensional framework, we were able to capture a broad range of social needs across multiple domains, providing a more nuanced understanding of their relationship with multimorbidity. This framework not only allows researchers to quantify and measure social needs in a systematic way, but it also provides a foundation for integrating social needs into clinical care. These findings will assist healthcare policymakers in developing more comprehensive care models that incorporate social needs, ultimately improving the management of multimorbidity and enhancing patient outcomes.

## Supplementary Information

Below is the link to the electronic supplementary material.

Supplementary Material 1

## Data Availability

Data may be obtained from a third party and are not publicly available. This study is based on Clinical Practice Research Datalink (CPRD) and is subject to a full license agreement that does not permit data sharing outside of the research team. However, data can be obtained by applying to CPRD (enquiries@cprd.com) for any replication of the study. The READ and SNOMED codes used are available in the Supplementary Material.
