# Supplementary material for: A multidimensional framework for mapping social need to electronic health records in people with multimorbidity
Source: Sci Rep. 2026 Jan 27;16:4629. doi: 10.1038/s41598-025-34881-9 (PMC12867966; doi:10.1038/s41598-025-34881-9)
Supplement: Supplementary file 1 — Supplementary Material 1 [file 41598_2025_34881_MOESM1_ESM.docx]

Supplementary Material

**Supplementary Table 1. List of Long-Term Conditions (LTC) identified in the Clinical Practice Research Datalink (CPRD) Database, mapped against the original 59 LTC conditions defined through national consensus.**

This table lists the 59 long-term conditions identified through national consensus and indicates whether each condition was available in the CPRD Gold and Aurum databases. Conditions were mapped using CPRD clinical code lists, with some conditions merged or renamed where CPRD recorded low counts.

|  | Long-Term Conditions in the Original 59  Conditions | Long-Term Conditions Present in  The Study |
| --- | --- | --- |
| 1 | Addison’s Disease | Yes |
| 2 | Anaemia | Yes  Present as Pernicious anaemia |
| 3 | Anxiety | Yes |
| 4 | Depression | Yes |
| 5 | Congenital Heart Disease | Yes |
| 6 | Chromosomal Abnormality | Yes |
| 7 | Bipolar Disorder | Yes |
| 8 | Schizophrenia | Yes |
| 9 | Chronic Liver Disease | Yes |
| 10 | Alcohol-related Liver Disease | Merged and renamed as: Chronic  Liver Disease and Alcoholic Liver  Disease |
| 11 | Aortic Aneurysm | Yes |
| 12 | Arrhythmia | Yes |
| 13 | Asthma | Yes |
| 14 | Autism | Yes |
| 15 | Bronchiectasis | Yes |
| 16 | Cancer | Yes |
| 17 | Cerebral Benign Tumours | No |
| 18 | Chronic Back Pain | No |
| 19 | Chronic Lyme Disease | Yes |
| 20 | Chronic Pancreatitis | Yes |
| 21 | Chronic Kidney Disease stage 3-5 | Yes |
| 22 | Chronic Pain | Yes |

| 23 | Chronic Obstructive Pulmonary Disease | Yes |
| --- | --- | --- |
| 24 | Chronic Urinary Tract Infections | Yes |
| 25 | Connective Tissue Disease | Yes |
| 26 | Coronary Heart Disease | Yes |
| 27 | Heart Failure | Yes |
| 28 | Hypertension | Yes |
| 29 | Cystic Fibrosis | Yes |
| 30 | Dementia | Yes |
| 31 | Diabetes | Yes |
| 32 | Drug or Alcohol Misuse | Yes |
| 33 | Eating Disorders | Yes |
| 34 | Endometriosis Adenomyosis | Yes |
| 35 | Epilepsy | Yes |
| 36 | Gout | Yes |
| 37 | Hearing Loss | Yes |
| 38 | Hemi/Para/Quadriplegia | No |
| 39 | HIV/AIDS | Yes |
| 40 | Inflammatory Bowel Disease | Yes |
| 41 | Long COVID | Yes  Present as Post-Acute COVID 19 |
| 42 | Meniere’s Disease | Yes |
| 43 | Multiple Sclerosis | Yes |
| 44 | Musculoskeletal Injury | Yes |
| 45 | Osteoarthritis | Yes |
| 46 | Osteoporosis | Yes |
| 47 | Parkinson’s Disease | Yes |
| 48 | Peptic Ulcer Disease | Yes |
| 49 | Peripheral Neuropathy | Yes |
| 50 | Peripheral Vascular Disease | Yes |
| 51 | Post-Traumatic Stress Disorder | Yes |
| 52 | Sickle Cell Disease | No |
| 53 | Stroke | Yes |
| 54 | Thyroid Disease | Yes |
| 55 | Transient Ischemic Attack | Yes |
| 56 | Tuberculosis | Yes |
| 57 | Valvular Diseases | Yes  Present as Heart Valvular Disorder |
| 58 | Venous Thromboembolism | Yes |
| 59 | Visual Impairment | Yes |

**Supplementary Table 2. READ and SNOMED CT codes used to identify 54 long-term conditions (LTCs) in the Clinical Practice Research Datalink (CPRD).**

This table provides the full list of codes (READ and SNOMED CT) used to define each of the 54 long-term conditions, as applied in the CPRD analysis.

[GitHub link with clinical codes](https://github.com/big-data-in-health/CPRD/blob/main/Medical_Codes/59LTC_CPRD.xlsx)

**Supplementary Table 3. Classification of CPRD-coded variables into eight derived social need domains.**

This table provides the full list of codes (Read and Medcode variables) used to define each domain of social need, as applied in the CPRD analysis. Domains include: Activities of Daily Living, Mobility Needs, Financial Needs, Disability Needs, Community Care Needs, Residential Status Needs, Social Care Networking Needs, and Bereavement Needs.

[GitHub link with social need codes](https://github.com/big-data-in-health/CPRD/blob/main/Medical_Codes/SOCIAL-NEED-CODES.xlsx)
